# The *Akebia* Genus as a Novel Forest Crop: A Review of Its Genetic Resources, Nutritional Components, Biosynthesis, and Biological Studies

**DOI:** 10.3389/fpls.2022.936571

**Published:** 2022-07-26

**Authors:** Ping Huang, Fengqi Zang, Changhong Li, Furong Lin, Dekui Zang, Bin Li, Yongqi Zheng

**Affiliations:** ^1^State Key Laboratory of Tree Genetics and Breeding, Key Laboratory of Forest Silviculture and Tree Cultivation, National Forestry and Grassland Administration, Research Institute of Forestry, Chinese Academy of Forestry, Beijing, China; ^2^Key Laboratory of State Forestry Administration for Silviculture of the Lower Yellow River, College of Forestry, Shandong Agricultural University, Tai'an, China

**Keywords:** *Akebia*, Lardizabalaceae, botany characteristic, nutrient composition, plant disease, fruit ripeness, status of resources

## Abstract

The genus *Akebia* belongs to the Lardizabalaceae family and comprises five species that are primarily distributed in East Asia. Plants of the *Akebia* genus comprise deciduous and semi-evergreen perennial twining vines that have been used in Chinese herbal medicine for at least 2000 years. The plants of this genus have the potential to form a novel forest crop with high nutritional and economic value because their fruit has a delicious sweet taste and rich nutrient components. In this study, we organized, analyzed, and evaluated the available published scientific literature on the botanical, ecological, and phytochemical characteristics of *Akebia* plants. Based on these studies, we briefly introduced botanical and ecological characteristics and focused on reviewing the development and utilization of wild genetic resources in the genus *Akebia*. We further explored the genus' rich nutritional components, such as triterpenes, flavonoids, polyphenols, polysaccharides, and fatty acids, and their potential use in food and health improvement applications. In addition, several papers describing advances in biotechnological research focusing on micropropagation, nutrient biosynthesis, and fruit ripeness were also included. This review provides comprehensive knowledge of the *Akebia* genus as a new forest crop for food and fruit utilization, and we also discuss future breeding and research prospects.

## Introduction

The domestication and cultivation of wild plant resources have encouraged the development of agricultural civilizations. Many success stories regarding the domestication of wild plants, such as corn, tomato, kiwifruit, and avocado, have significantly promoted population growth and social and economic development (Huang H. W. et al., 2021). Forest plants play a vital role in supporting agricultural production and in alleviating poverty, particularly in developing countries by providing food and herbal medicines (Beardmore et al., 2014). Although the successful domestication and cultivation of woody plants is a long and difficult process, it remains an important solution to many issues regarding human nutrition, health, and safety.

The genus *Akebia* belongs to the Lardizabalaceae family of flowering plants and is widely distributed throughout East Asia, such as in China, Korea, and Japan. *Akebia* plants comprise perennial deciduous vines that produce purple flowers and edible fruits. These plants have provided raw materials for traditional Chinese herbal medicine for thousands of years, and recently, they have also been recorded in the American Herbal Pharmacopeia and European Pharmacopeia (Upton, 2016; Pharmacopoeia of the People's Republic of China, 2020; Council of Europe convention on the elaboration of a European pharmacopoeia., 2021). *Akebia* fruit is commonly known as “wild banana” or “northern banana” in China, and local people in East Asia praise it for its delicious taste. In addition, the common name for *Akebia* plants is “chocolate vine,” and some species have been introduced and cultivated in Europe and the United State because of the plant's attractive and fragrant flowers. Many studies have focused on the phytochemistry, experimental pharmacology, and clinical pharmacology of the *Akebia* genus, including the isolation and purification of medicinally active ingredients and their biological activity (Liu et al., 2018; Maciag et al., 2021). However, recent studies have indicated that *Akebia* plants offer valuable options as horticultural crops because of their health and nutritional benefits and ornamental traits.

The present study aimed to review the existing scientific literature on the genus *Akebia*, which could be developed as a new horticultural crop, with a particular emphasis on fresh fruit and oil production, and ornamental use. We summarize the available information on botanical and ecological characteristics, the status of wild genetic resources, cultivation and breeding, plant diseases, main uses, and corresponding phytochemicals. In addition, recent advances in biotechnology research are reviewed, with particular attention being given to micropropagation technology, genomics, and multi-omics analysis of key metabolite biosynthesis, as well as biochemical and molecular biological processes of fruit cracking and softening.

## The *Akebia* Genus in General

The *Akebia* genus belongs to the family Lardizabalaceae, which comprises nine genera (*Sargentodoxa, Decaisnea, Sinofranchetia, Archakebia, Akebia, Stauntonia, Holboellia, Boquila, Lardizabala*). A large proportion of species in the Lardizabalaceae are native and widely distributed in Asia (from the Korean peninsula to the Indochina peninsula), while only two genera (*Boquila, Lardizabala*) occur in South America (Kofuji et al., 1994; Qin, 1997; Christenhusz, 2012; The Plants List, 2020). Previously, more attention had been given to the *Akebia* genus because of its broader distribution and historic resource utilization in Asia. According to the world flora online database, dozens of species have been recorded from the *Akebia* genus; however, only five species have been accepted and described: *Akebia chingshuiensis* (T. Shimizu), *A. longeracemosa* (Matsum), *A. quinata* (Houtt.) Decne, *A. trifoliata* (Thunb.) Koidz, and *Akebia* × *pentaphylla* (Makino) Makino. Three *Akebia* subspecies are also present in *A. trifoliata* (subsp. *longisepala*, subsp. *trifoliata*, and subsp. *australis*) (Wu et al., 2013). These plants are woody deciduous and sub-evergreen climbers, which are monoecious and have twining stems, while their blades comprise palmately compound leaves with long petioles either in an alternate arrangement or are clustered on short branches. Five *Akebia* species are distributed in China, Japan, and Korea, and two of these species are endemic to China (*A. chingshuiensis* and *A. longeracemosa*). *Akebia quinata* and *A. trifoliata* are the most popular plants used in traditional medicines. Besides being a source of Chinese herbal medicine, *Akebia* plants are also grown as ornamental crops in China or garden plants in Europe and the United States (Li et al., 2010).

## Botanical and Ecological Characteristics of the *Akebia* Species

**Figure 1:**
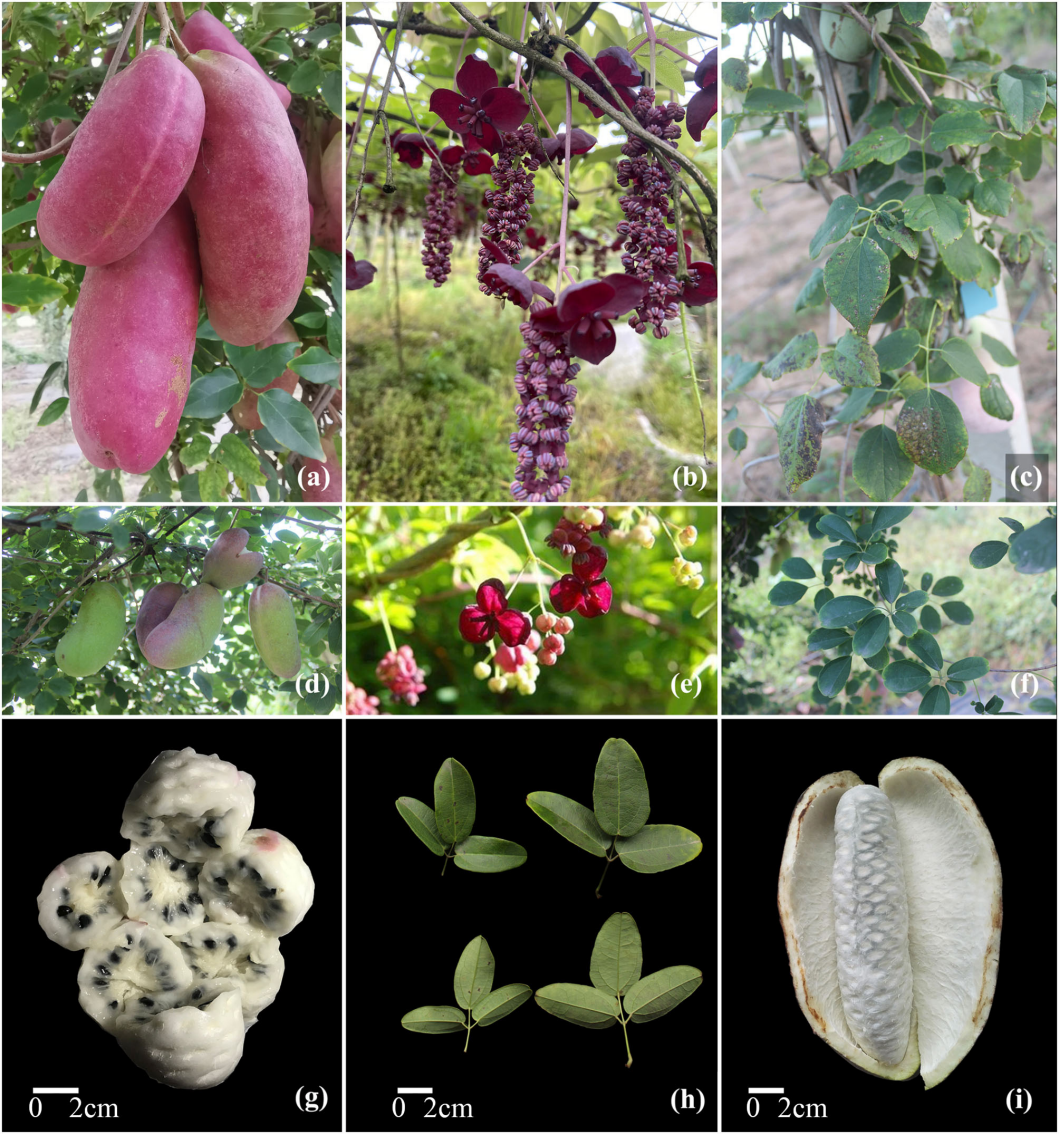
*Akebia* trifoliata and *A. quinata*. **(a–c)** fruit, flower, and leaves of *A. trifoliata*; **(d–f)** fruit, flower, and leaves of *A. qui*nata. **(g–i)** flesh, leaflet, and cracking fruit of *A. trifoliata*.

*Akebia quinata* was first described in 1779 by Maarten Houttuyn, a Dutch naturalist. The herbarium was collected in Japan and originally named *Rajania quinata*. The name *A. quinata* was officially described in 1845, and this species was identified as a generitype of the *Akebia* genus (Christenhusz and Rix, 2012; Wu et al., 2013). *Akebia quinata* (common name: chocolate vine or five-leaf *Akebia*) is a deciduous twining woody climber. The compound leaf of this species is bluish-green with five leaflets (Figure 1f). The species is monoecious, and each inflorescence includes one or two female flowers (2.5 cm in diameter) and four to ten male flowers. Female flowers (dark purple) are twice as large as male flowers (pale pink with pinkish-purple stamens) (Figure 1e). Both flowers have a sweet scent but contain no nectar, the petals are absent, and the sepals (white to pale purple) serve the purpose of attracting pollinators (Kawagoe and Suzuki, 2003). The observed non-randomness in flower choice by pollinators may increase the probability of cross-pollination and lower the risk of geitonogamous pollination owing to floral sexual dimorphism (Kawagoe and Suzuki, 2002). Flowering occurs from April to May, and female flowers open several days earlier than male flowers within the inflorescence. However, anthesis of male and female flowers overlaps within inflorescences, suggesting that self-pollination is still possible, while geitonogamous self-pollination has been shown to significantly reduce outcrossed seed production (Kawagoe and Suzuki, 2005). The fruit of *Akebia quinata* is a fleshy follicle, purple at maturity, straight or slightly incurved and oblong to ellipsoid in shape, and approximately 3–4 cm in diameter and 5–8 cm long (Figure 1d). Each fruit has over 100 brown to black shiny seeds with an ovoid, oblong shape, and edible white pulp. The natural distribution of *A. quinata* comprises mountainous slopes at altitudes between 300–1500 m in China, the Korean peninsula, and Japan. Habitat selection generally comprises forest ecotones along streams, or scrub on mountain slopes, and the species generally prefers fertile and sufficiently moist soils which provide resistance to cold (Christenhusz and Rix, 2012).

The history of the cultivation and domestication of *A. quinata* is brief; however, several varieties have been bred and developed. Ornamental traits such as a particular flower or leaf colors may be selected for later selective breeding. For example, *A. quinata* “Alba” is a variety with pale green stems and small white flowers, with leaves that turn bright yellow in the fall. Similarly, *A. quinata* “Shiro Bana” has pale yellow to white flowers, large yellow-green leaves, and dark purple fruit; *A. quinata* “Silver Bells” has creamy-white male flowers with dark purple stamens and pink female flowers. *Akebia quinata* “Rosea” has mauve-pink flowers that are paler than typically observed, which is beneficial since it provides a contrast against the dark green foliage in which it occurs. *Akebia quinata* “Variegata” is a unique variety of the species with white and green splashes on its foliage, which poses as an attractive backcloth for pink-purple inflorescences. All the above varieties were developed and grown under warm conditions.

Another species worth noting is *A. trifoliata*, commonly named the “Three-leaf *Akebia*.” This species has a large number of synonymous Latin names in the plant list database (The Plants List, 2020). This species was first described in The Botanical Magazine, Tokyo (Bot. Mag.) in 1925. This species has three subspecies in the updated Flora of China, and their varied traits are described in Table 1, including the shape and size of sepals in male and female flowers and the shape of the leaflet margin (Wu et al., 2013). *Akebia trifoliata* is a monoecious, deciduous, and twining woody climber. Its leaves are palmately compound, with three ovate, ovate-oblong, or broadly ovate leaflets with wavy margins, which are slightly larger and brighter than those of *A. quinata* (Figures 1c,h). The flowers are monoecious, the axillary racemes grow on short branches and contain 15–30 male flowers, which have three pale purple to purple sepals and six pinkish-purple stamens, and one to two female flowers with three purplish brown, dark purple, or purplish-black sepals and four to nine carpels (Figure 1b). Both flowers have slight cinnamon scents, and the flowering period occurs from April to May. The fruit is oblong, straight, or slightly curved, and the fruit color changes from yellow to brown or from pale purple to bluish as it ripens (Figure 1a). The fruit is approximately 4–5 cm in diameter and 8–15 cm in length, which is larger than that of *A. quinata* (Figure 1i). Each fruit also has numerous brown to black shiny seeds with an ovoid, oblong shape, and white flesh (Figure 1g).

**Table 1:** Natural distribution and botanical characteristics of *Akebia* species.

**Species**	***A. quinata***	***A. trifoliata***			***A. × pentaphylla***	***A. chingshuiensis***	***A. longeracemosa***
**Subspecies**		**Longisepala**	**Trifoliata**	**Australis**			
Altitude(m)	300–1500	600–800	200–2000	300–2100	-	1500–2400	300–1500
Distribution	China (West of the provinces of Sichuan and Hubei), Japan (Honshu, Kyushu, Shikoku) and Korea	China (Southeast the province of Gansu)	China (Central provinces), Japan (Hokkaidô, Honshu, Kyushu, Shikoku).	China (South provinces)	Japan	China (Taiwan)	China (South provinces)
No. of leaves	(3–)5(−7) leaflets.		3(−5) leaflets		(3)-5 leaflets	3(−5) leaflets	(3–)5(−7) leaflets.
Shape of Leaflets	Leaflets papery abaxially glaucous	Usually sinuate-dentate;			Margin with slightly wavy edges	Margin subentire	Margin entire, sub-leathery, leaflets, abaxially pale green
		Margin subentire, leathery, base truncate to cuneate	Margins sinuate to shallowly lobed, leaflets papery to sub-leathery	Leaflets leathery, margin usually entire, very rarely irregularly sinuate			
Sepals of male flowers	6–8 mm	2–3 mm			-	1–2 mm	4–4.5 mm
Shape of sepals of male flowers	Occasionally pale green or white, broadly cucullate-ovate, 6–8 × 4–6 mm	Oblong, more than 2 × as long as stamens	Elliptic to broadly elliptic, ± as long as stamens	Elliptic to broadly elliptic, ± as long as stamens	-	Elliptic, navicular, 1–2 mm, glabrous	Elliptic-oblong to broadly elliptic, 4–4.5 × ca. 3 mm
Racemes	Racemes 6–12 cm, 4–8(−11)-flowered	Racemes 6–16 cm			Racemes 6–16 cm	Racemes ca.10 cm	Racemes 12–18 cm, 23–35(−43)-flowered
Flowering time	Apr–May	Apr	Apr–May	Apr–May	Apr–May	Apr–May	Mar–Apr,
Fruit	Fruit purplish at maturity, straight or slightly incurved, oblong to ellipsoid, 5–8 × 3–4 cm.	–	Fruit grayish white and slightly pale purple at maturity, oblong, 6–8 × 2–4 cm.	Fruit yellowish brown at maturity, 6–8 × 3–5 cm.	Fruit purple at maturity, 5–10 cm	–	Fruit solitary or paired, reddish purple at maturity, oblong, 6–7 × ca. 2 cm
Fruiting time	Jun–Aug	–	Jun–Sep	Jun–Sep	–	–	Aug

*Data sourced from Flora of China (http://www.iplant.cn/info/Akebia?t=foc), GRIN-Global (https://npgsweb.ars-grin.gov/gringlobal/taxonomylist?category=species&type=genus&value=Akebia&id=345) and Plants of World Online (http://www.plantsoftheworldonline.org/taxon/urn:lsid:ipni.org:names:3933-1)*.

*Akebia trifoliata* has a broader natural distribution range in East Asia than that of *A. quinata* from subtropical to temperate regions. Its natural habitats are similar to that of *A. quinata*, and the species exhibits a preference for clay soils that retain moisture under semi-shaded conditions (Li et al., 2010). *Akebia trifoliata* ssp. trifoliata is naturally distributed in central China, with an increase in density along the Qin-Ling mountain range. *Akebia trifoliata* subsp. australis occurs widely in southern China, from south of the Yangtze River to the center of the island of Taiwan. *Akebia trifoliata* subsp. longisepala is distributed in a small southeastern region of the Gansu Province, China. *A. trifoliata* is a popular ornamental horticultural plant worldwide. The number of *A. trifoliata* varieties appears to be lower than that of *A. quinata*. The variations of ornamental traits among *A. trifoliata* comprise leaves with white markings or spots (*A. trifoliata* “Shunjitsu”, *A. trifoliata* “Shiromi,” *A. trifoliata* “Aoba”), larger fruit (over 15 cm long, *A. trifoliata* “Big fruit”), and purple-black intensely fragrant flowers (*A. trifoliata* “Deep Purple”).

*A. longeracemosa* has longer racemes, smaller flowers, and slender leaflets and branches. Previous studies have reported two varieties of *A. longeracemosa*, namely, *A. longeracemosa* “Giganteiflora” and *A. longeracemosa* “Victor Secret.” The former has a bigger flower than the original species, and the latter's leaflet has a light green speckle present. *Akebia chingshuiensis* has a narrow distribution range when compared to *A. trifoliata*, and small sepals are present on male flowers (1–2 mm) while a subentire leaflet margin is present (Wu et al., 2013) (Table 1). *Akebia* × *Pentaphylla* is an accepted name of a species in the genus *Akebia* (family Lardizabalaceae), described in Bot. Mag. in 1902. It is considered to be a naturally occurring hybrid between *A. quinata* and *A. trifoliata*. The typical morphology of *A*. × *pentaphylla* has five leaflets with slightly wavy edges and pale purple male and female flowers (Makino, 1989; Satake et al., 1997). However, some naturally occurring individuals of this species exhibit boundary morphologies (Kitaoka et al., 2009). Two varieties of this species have been published: *Akebia* × *pentaphylla* “Pikapika” is a variety with white young leaflets and green branches; *Akebia* × *pentaphylla* “Purple Incense” has fragrant dark purple flowers and exceptionally large fruit.

## Main Uses and Phytochemical Components

### Traditional Herb

*Akebia* plants have been used as a traditional Chinese herb for thousands of years. *Akebiae Caulis* (Mu Tong) and *Akebiae fructus* (Yuzhizi) are two types of traditional Chinese herbs that were recorded in the Pharmacopoeia of the People's Republic of China (2020). Both come from processing stems and seeds, respectively, and are related to *A. quinata* and *A. trifoliata*. The Pharmacopeia states that *Akebiae Caulis* has therapeutic effects which assist in the treatment of diuresis, nervousness, and analgesia, and *Akebiae fructus* is used to treat indigestion and abdominal pain. The stems and fruits of *A. quinata* were also recorded in the Japanese Pharmacopeia in 2001 (Leung et al., 2006). In addition, the stems of *A. trifoliata* were listed in the American Herbal Pharmacopeia in 2016 (Upton, 2016). In Europe, only the stem (*Akebiae Caulis*) is officially considered a useful raw material in the 9th edition of the European Pharmacopeia. In these Pharmacopeias, the raw materials of herbal medicines must be standardized to contain specific phytochemical components. Example oleanolic acid (> 0.15%), calceolarioside B (> 0.15%), and α-hederin (> 0.20%), which were grouped as triterpenoid saponins, triterpenoids, and phenylpropanoids, respectively.

**Figure 2:**
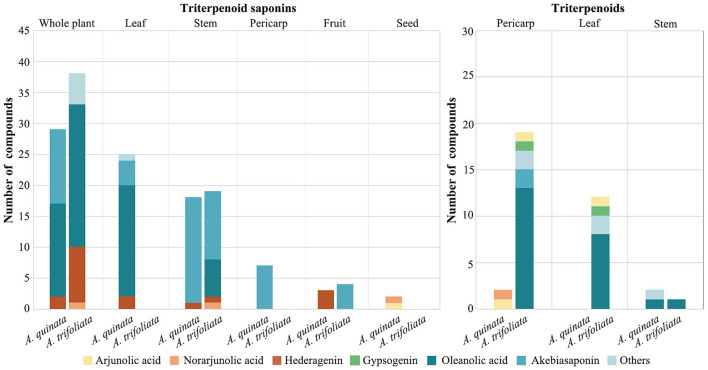
Species and distribution of triterpenoid saponins and triterpenoid in *Akebia* plants. The statistics are based on a phytochemical review of the *Akebia* genus (Maciag et al., 2021).

Triterpenoids are biosynthesized in plants through the cyclization of squalene, a triterpene hydrocarbon, and a precursor of all steroids (Phillips et al., 2006). They can be further subdivided into diverse groups (Petronelli et al., 2009). Increasing evidence indicates that triterpenoids have broad-spectrum pharmacological activities, coupled with a low toxicity profile (Setzer and Setzer, 2003; Wang et al., 2015). In a previous study, four types of triterpenoids were isolated from the stems (betulin and 2α,3α,23-trihydroxyoleane-12-en-28-oic acid) and pericarps (arjunolic acid and norarjunolic acid) of *A. quinata* (Higuchi and Kawasaki, 1976). Numerous triterpenoids have been found among various parts of the *Akebia* plant. When compared with *A. quinata*, a total of 32 kinds of triterpenoids are present, isolated from leaves, stems, and pericarps of *A. trifoliata*, such as arjunolic acid, gypsogenic acid, mesembryanthemoidigenic acid, serratagenic acid, stachlic acid A, akebonoic acid, and oleanolic acid (Maciag et al., 2021). The triterpenoid compounds in the pericarp and leaves of *A. trifoliata* were more diverse than those of *A. quinata* (Figure 2). In *A. trifoliata*, triterpenoids have only been found in the fruit and leaves, while oleanolic acid and akebiasaponins have been isolated from the stems (Liu et al., 2018) (Figure 2).

Triterpene saponins are triterpenes that belong to saponin compounds. They are composed of a triterpene aglycone linked to one, two, or three saccharide chains of varying size and complexity. These compounds have various biological and pharmacological activities, such as antimicrobial activity, diuretic action, and promotion of cholesterol metabolism (Hostettmann and Marston, 1995; Vincken et al., 2007; Thimmappa et al., 2014). In *Akebia* plants specifically, triterpene saponins, one of the most diverse compounds, accumulate in high concentrations in the stems, fruits, and seeds (Ochmian et al., 2014; Liu et al., 2018). The triterpene aglycones of *Akebia* plants include oleanlic acid, norarjunolic acid, arjunolic acid, and hederagenin (Figure 2). The triterpene saponin profile of *Akebia* plants, based on previous reports, suggests that there are significant differences in the composition of these compounds in the stems of *Akebia* species; for example, mutongsaponin C and saponin P1 were only found in the stems of *A. trifoliata* and its subsp. *australis* (Gao and Wang, 2006). Some rare types of triterpene saponins in plants have been isolated and identified in the stems of *A. trifoliata*, such as akemisaponins B-F, which are triterpene saponins of the 30-norolean-20(21)-ene type (Iwanaga et al., 2012).

### Food and Nutrition

Human exploration and utilization of wild plant resources have an extensive history. *Akebia* plants have historically been eaten as wild fruit in East Asia, especially those sourced from *A. quinata* and *A. trifoliata*. The latter fruits are sweet and juicy, and are rich in nutritional components, such as sugars, proteins, amino acids, vitamins, and mineral elements, in addition to medicinal uses, suggesting that it has the potential to develop into a novel forest fruit crop (Li et al., 2010). Therefore, numerous studies have focused on the nutritional components of the edible parts of *Akebia* plants. In *A. trifoliata* fruit, the total sugar and reducing sugar contents could reach up to 14.9 g/100 g and 10.2 g/100 g, respectively, and the high reducing sugar content and low acidity make their flesh taste sweet (Li and Li, 1991). In addition, there are various vitamins in the flesh of the fruit, such as β-carotene and vitamins B and C, which vary from 108 to 930 mg/100 g DW, which is higher than that of apples, grapes, and bananas (Liu and Qian, 2002; Li et al., 2010). The fruit flesh of *A. quinata* and *A. trifoliata* are rich in mineral elements, amino acids such as potassium (3.2–4.9 mg/g), magnesium (1.00–1.51 mg/g), and calcium (0.47–0.49 mg/g), with a total amino acid amount of 818.5 mg/100 gFW which contains an additional eight essential amino acids (Liu and Qian, 2002; Zhang et al., 2003). This suggests that the *Akebia* plant has the potential to provide novel fruits that are nutritionally rich. In addition to the flesh, the pericarp is an important source of plant nutrients, which can be developed for fruit beverages or teas (Table 2).

**Table 2:** Main nutrient components in *Akebia* plants.

**Species**	**Parts of plant**	**Nutrition**	**Compounds**	**Content**	**References**
*A. trifoliata*	Fruit (flesh)	Sugar	Total sugar	14.9 g/100 g FW	Liu and Qian (2002)
			Reducing sugar	10.2 g/100 g FW	Liu and Qian (2002)
			Fructose	4.10 g/100 g FW	Li et al. (2010)
			Glucose	2.78 g/100 g FW	Li et al. (2010)
				70–361 mg/g FW	Li et al. (2021)
			Sucrose	1.57 g/100 g FW	Li et al. (2010)
			D-mannose	11–19 mg/g	Li et al. (2021)
		Vitamin	Vitamin C	108–930 mg/100 g FW	Liu and Qian (2002); Wang et al. (2004)
			Vitamin PP	0.51% w/w	Wang et al. (2004)
			Vitamin B1	0.23% w/w	Wang et al. (2004)
			Vitamin B2	0.41% w/w	Wang et al. (2004)
			B-carotene	0.052% w/w	Wang et al. (2004)
		Protein	Crude protein	1.07 g/100 g FW	Liu and Qian (2002)
				8.16% ± 0.11% FW	Li et al. (2021)
			Total amino acid	818.5 mg/100 g FW	Liu and Qian (2002)
		Mineral element	Potassium	340–496 mg/100 g FW	Zhang et al. (2003)
			Magnesium	100–151 mg/100 g FW	Zhang et al. (2003)
			Calcium	47–48 mg/100 g FW	Zhang et al. (2003)
			Zinc	1.92–2.47 mg/100 g FW	Zhang et al. (2003)
			Iron	0.83–1.00 mg 100 g FW	Zhang et al. (2003)
	Fruit(pericarp)	Sugar	Total sugar	32.61% ± 0.18% w/w	Zhang et al. (2022)
			Reducing sugar	19.31% ± 0.21% w/w	Zhang et al. (2022)
			Pectin	20.08% ± 0.20% w/w	Zhang et al. (2022)
		Protein	-	8.16 ± 0.11% w/w	Zhang et al. (2022)
		Total flavonoids	-	20.58 ± 0.12 mg/g DW	Zhang et al. (2022)
		Total polyphenolics	-	45.20 ± 0.18 mg/g DW	Zhang et al. (2022)
	Seed	Oil		30.2–48.8% w/w	Su et al. (2021)
		Fatty acid	Oleic acid	155.9–261.5 mg/g, 36.6–45.2%	Su et al. (2021)
			Linoleic acid	113.8–156.4 mg/g, 23.5–30.8%	Su et al. (2021)
			Palmitic acid	101.8–149.1 mg/g, 20.3–25.7%	Su et al. (2021)
		Protein	Total protein	17.23% w/w	Du et al. (2012)
			Albumin	51.65% w/w	Du et al. (2012)
			Glutelin	46.40% w/w	Du et al. (2012)
	Flower	Anthocyanin	Pelargonidin-3-O-arabinoside	*-*	Jiang et al. (2020)
			Cyanindin-3-O-glucoside	*-*	Jiang et al. (2020)
			Peonidin 3-galactoside	*-*	Jiang et al. (2020)
			Delphinidin-3-O-arabinoside	*-*	Jiang et al. (2020)
			Delphinidin-3-O-di-hexoside	*-*	Jiang et al. (2020)
			Cyanidin-3-O-di-hexoside	*-*	Jiang et al. (2020)
			Delphinidin-3-O-rutinoside	*-*	Jiang et al. (2020)
			Cyanidin 3-O-(6″ acetyl) glucoside	*-*	Jiang et al. (2020)
			Cyanidin-3-O-(p-coumaroyl) rutinoside	*-*	Jiang et al. (2020)
*A. quinata*	Fruit	Essential oil	Limonene	*-*	Kawata et al. (2007)
			Eugenol	*-*	Kawata et al. (2007)
			Octanal	*-*	Kawata et al. (2007)
			P-cymene	*-*	Kawata et al. (2007)
	Stem	Essential oil	Hexanoic acid	*-*	Kawata et al. (2007)
			Palmitic acid	*-*	Kawata et al. (2007)
			(2E, 4E)-decadienal	*-*	Kawata et al. (2007)
			Hexanol	*-*	Kawata et al. (2007)
	Fruit (Flesh)	Mineral element	Potassium	321 mg/100 g FW	Zhang et al. (2003)
			Magnesium	100 mg/100 g FW	Zhang et al. (2003)
			Calcium	49 mg/100 g FW	Zhang et al. (2003)
			Zinc	2.1 mg/100 g FW	Zhang et al. (2003)
			Iron	3.21 mg 100 g FW	Zhang et al. (2003)

*FW, Fresh weight; DW, Dry weight; w/w, weigh/weigh*.

Vegetable oil, extracted from a variety of plants, primarily sourced from the seeds of plants, is a common ingredient in many diets worldwide. Several previous reports have found high oil contents in the seeds of *A. trifoliata* (30.2–48.8%) which also has a specific fatty acid profile, containing oleic acid (155.9–261.5 mg/g, 36.6–45.2%), linoleic acid (LA, 113.8–156.4 mg/g, 23.5–30.8%), and palmitic acid (PA, 101.8–149.1 mg/g, 20.3–25.7%). The ratio of saturated, monounsaturated, and polyunsaturated fatty acids is 1:1.5:1, which meets the World Health Organization's recommended standards for edible oil (Su et al., 2021). Another main nutrient found in the seed is protein, and its main components are albumin and glutelin (Du et al., 2012).

### Ornamental Gardening and Others

*Akebia* is a suitable garden plant with dainty scented spring flowers and cute palmate leaves. As aforementioned, a series of varieties have been developed and bred from wild plants. The major variation in characters includes flower color (white, yellow, pink, light purple, and dark purple), leaf color, fruit color, and fruit size, which make these plants more popular than other wild species and are better suited as ornamental plants. Previous studies have reported that flower color is mainly affected by anthocyanins, specifically the type and content, and a group of flavonoids (Zhao and Tao, 2015). Earlier studies have also reported the presence of polyphenols, including anthocyanins, phenolic acids, and flavonols in the flowers of *A. quinata* (Tang and Eisenbrand, 1992). The variation in flower color in *Akebia* plants also indicates the diversity of anthocyanins. A recent study has suggested that the main types of anthocyanins found in the flowers of *A. trifoliata* include Cyanindin (Cy), Pelargonidin (Pg), Peonidin (Pn), and Delphinidin (Dp). The crude yield of anthocyanins could reach up to 50.87 mg C3G/100 g in flower powder (Jiang et al., 2020). These diverse types of anthocyanins are suggestive of the high breeding potential of *Akebia* as garden plants.

In addition to the main uses listed above, substances extracted from *Akebia* plants may have other uses. The volatile substances in the stems and fruits of *A. quinata* have different composition profiles when compared to the essential oils; the main components of essential oil from the stem are monoterpenoids and saturated. However, in the fruit, the main components comprise a high concentration of saturated fatty acids and unsaturated short-chain aldehydes (Kawata et al., 2007). Additionally, there is a high concentration of pectin in the pericarp, which is typically regarded as waste. Utilizing this waste could be another important way to use plant resources efficiently. Pectin extracted from *A. trifoliata* pericarps was developed and processed into a new medical sponge which could effectively accelerate the healing of infected wounds because of its good water solubility and high galacturonic units (Yu et al., 2019). Another study indicated that pectin from *A. trifoliata* pericarps could be used as a wall material to coat curcumin-loaded zein nanoparticles (Cai et al., 2020). These reports indicate that natural pectin from *A. trifoliata* pericarp waste represents a promising green macromolecule for utilization in the pharmaceutical and food industries.

### Analysis and Assessment of Genetic Resources

The cultivation and domestication of plants is not only the source and foundation of human civilization but also a natural solution to major problems such as food security and nutrition improvement. The increase in yield and the improvement in quality of horticultural plants are distinct from the species richness and abundant intraspecific genetic diversity of wild sources, which provides the material basis for new varieties (This et al., 2006; Bai and Lindhout, 2007). Phenotypic diversity is mainly composed of growth traits, yield traits, resistance traits, and quality traits, and the phenotype of these traits is dependent on genetic components and plant-environment interactions which provides further potential for genetic improvement (Deikman et al., 2012). The wild genetic resources of the *Akebia* genus are abundant but less developed. Previous studies on the analysis and evaluation of genetic resources have primarily focused on the assessment of genetic diversity, growth traits, yield traits, and quality traits of wild resources in the *Akebia* genus.

### Assessment of Genetic Diversity

Genetic diversity is essential for biodiversity conservation and plant breeding because it can give rise to diverse physical trait attributes and the capacity to adapt to stress, diseases, and environmental changes (Tester and Langridge, 2010). New varieties of plants can be grown by crossbreeding different genetic variants for desirable traits. A large variety of horticultural crops originated and were domesticated from wild genetic sources, such as apples and roses (Cornille et al., 2014; Raymond et al., 2018). Knowledge of the pattern of genetic diversity within wild plant species may contribute to revealing their evolutionary history and assessing their evolutionary potential, which forms the basis for breeding and genetic improvement. Hence, previous studies have focused on the development of tools for the genetic analysis of the *Akebia* genus, for example, genomic SSR and EST-SSR (Li et al., 2009; Niu et al., 2019), which are effective tools for genetic diversity analysis and the assessment of plants. In fragmented habitats, the genetic diversity of three *Akebia* species, including *A. trifoliata* ssp. australis, *A. trifoliata* ssp. trifoliata, and *A*. *quinata*, were assessed using eight genomic SSR markers, and the results showed a relatively high genetic diversity (mean H_e_ > 0.6) and low levels of genetic differentiation among populations. These results may be attributed to the outcrossing mating systems, lack of long-distance seed dispersal, and persistent seed banks of *Akebia* species (Li et al., 2019). Recently, another report focused on the genetic diversity of *A*. *trifoliata* germplasms collected *ex-situ* from GenBank. A set of core germplasm collections were screened, and the results indicated that the collected germplasms comprised moderate genetic diversity. This could help to improve the efficiency of conservation and breeding (Zhong et al., 2021).

### Phenotypic Variation of *Akebia* Genus

Phenotypes comprise a series of observable or measurable characteristics of individuals resulting from their genotype and interaction with the environment. These characteristics include morphology, growth, yield, quality, and resistance. After a long process of evolution, natural selection, and artificial domestication, wild forest plants obtain rich genetic and phenotypic variation, providing an abundant material basis for the development and utilization of genetic resources (Huang, 2009). Previous phenotypical analyses and the assessment of genetic resources in *Akebia* plants focused on economic and quality traits. The studies also investigated phenotypic variations among the economic characteristics of *Akebia* spp., including fruit economic characteristics, fruit flesh nutritional components, rattan medicinal components, seed oil content, and fatty acid composition, suggesting that genetic resources from different geographical provenances could be utilized for different purposes, for example, fresh fruit, oil production, or herbal medicine. Fruit traits are vital for economically important forest species, and the development and use of genetic resources are dependent on the economic characteristics of the fruit. Understanding the genetic variation and hereditability of fruit traits could help develop a high-efficiency breeding strategy to achieve maximum genetic gain. Zou et al. (2018) identified 11 key traits that have high repeatability and a wide phenotypic correlation in *A. trifoliata* and used the Smith–Hazel index to develop index coefficients to identify superior genotypes associated with increased single fruit weight (SFW), an edible ratio (ER), and decreased seed number (SN). This is expected to enhance the selection of an excellent fruit-product genotype. However, the domestication and selective breeding of *A. trifoliata* for a variety of fruits are still in early development.

Based on the physicochemical properties and fatty acid components of seed oil, a few germplasms of *A. trifoliata*. were screened through multilocation testing, which may have the potential to breed varieties for producing edible oil (Li et al., 2020). A more comprehensive genetic and agro-climate variability in the seed fatty acid profile of *A. trifoliata* in China was investigated, and the results indicated that the oil content of seeds was strongly influenced by the geographic environmental variation and germplasms. The oil content of seeds from different provenances was variable but high (30.2–48.8%), and the variation in their fatty acid profiles was significant. It has also been proposed that the species distribution of *A. trifoliata* around 35°N is more desirable for commercial cultivation for vegetable oil production, because of the much cooler and drier climate, along with larger diurnal temperature fluctuations which are beneficial to oil accumulation in *A. trifoliata* seeds (Su et al., 2021).

### Plant Disease in *Akebia* Genus

Plant diseases not only significantly affect the normal growth, development, survival, and reproduction of plants but also negatively affect the production of horticultural crops. These include reducing yields and quality, accumulating toxins, shortening the storage period, and affecting the transportation of crops (Chakraborty and Newton, 2011). With the development and domestication of *Akebia* plants, their cultivation area is also gradually increasing, and the problem of disease in the growth period is becoming increasingly prominent. To date, the main plant diseases reported in the genus *Akebia* include leaf spots, anthracnose, powdery mildew, and brown spots which have been isolated and identified, mainly involving *Alternaria tenuissima, Phytophthora nicotianae, Corynespora cassiicola*, etc. Symptoms of these diseases are presented in Table 3. These plant diseases can result in reduced photosynthesis in leaves, the rotting of stems, discoloration of the pericarp, shrinkage, and the premature drop of fruit. These diseases also lower the fruit quality and horticultural value in practical production. Very few studies have focused on disease resistance and control in *Akebia* plants, and suggested the use of chemical agents, such as ammonium sulfate and ammonium chloride, in addition to biological antagonists (*Bacillus subtilis*) that can effectively inhibit the growth of pathogenic bacteria in the laboratory (Zhang et al., 2015, 2019). However, fundamental research on disease pathogenesis and microbial-plant interaction mechanisms has not been conducted yet.

**Table 3:** Main reported plant diseases in *Akebia* plants.

**Disease**	**Species**	**Phytopathogen**	**Symptoms**	**Incidence**	**Period**	**References**
Powdery mildew	*A. quinata*	*Microsphaeria akebiae*	-	**-**	October	Scholler and Gams (1998).
		*Oidium* sp. subgenus Pseudoidium	The upper surfaces of leaves were covered with white mycelium, and the corresponding abaxial surface of infected leaves were chlorotic. Young, green stems also affected showed extended chlorosis. As the disease progressed, infected leaves turned yellow and died.		Summer	Garibaldi et al. (2004)
Leaf spot	*A. trifoliata*	*Corynespora cassiicola*	Initial symptoms consisted of small (less than 5 mm in diameter), circular, purple-brown leaf spots. Spots later enlarged and became elliptical, circular, or irregular with gray-white centers and dark brown rims. The centers were slightly concave. The spots could coalesce with each other, resulting in leaf desiccation and wilting.	80%	July	Ye et al. (2013)
		*Alternaria tenuissima*	Initial symptoms consisted of small rufous leaf spot and enlarge and became to 1–2 mm; then became elliptical, circular, or irregular disease spot; finally, the spots could coalesce with each other, resulting in leaf desiccation and wilting.	53%	April to September	Liu et al. (2013)
		*Phytophthora nicotianae*	Small brown spots, subcircular or irregular-shaped brown necrotic lesions. In severe cases, the leaves became completely necrotic and abscised.	30–40%	July	Cheng et al. (2020)
Anthracnose	*A. trifoliata*	*Colletotrichum gloeosporioides*	Diseased leaves exhibited irregular gray-brown spots with dark brown edges and dark brown. As disease progressed, white mycelium appeared on stems, causing stem rot and fruit drop. Several round or needle-shaped dark brown spots formed on fruit peel, coalescing into irregular, slightly sunken blotches. Under high humidity, the whole fruit turned brown, and the spots were covered by white mycelia, greatly affecting the fruit's ornamental quality.	up to 15%	December to May the following year	Pan et al. (2021)
		*Colletotrichum acutatum*	Initial symptoms appeared as small necrotic brown spots, 1–2 mm diameter, on the leaf margin, central vein, and petiole. As the disease progressed the lesions expanded and coalesced, and the center of the lesions turned grayish white. Severely diseased leaves wilted and fell off. In humid conditions, acervuli containing orange to salmon-pink masses of spores emerged on lesions	-	October	Kobayashi et al. (2004)
		*Nigrospora sphaerica*	The infected fruits were shrunken, colored dark brown, and withered to death.	10%	-	Hong et al. (2021, 2022)

### Modern Biotechnology Progress in *Akebia* Genus

#### Tissue Culture

Plant tissue culture comprises a cell engineering technique that involves excising explants and growing them on media. This technique has a wide range of uses, such as the rapid propagation of virus-free and haploid plants, while it also offers a valuable tool for research in plant science (Kumar and Loh, 2012). A series of tissue culture methods have been established for *A. quinata* and *A. trifoliata* (Table 4). An earlier study focused on callus cultures of *A. quinata* to assess secondary metabolites, and the results indicated that a group of triterpene saponins was isolated from the biomass of the callus cultures (Ikuta, 1991). A bulk of existing research focuses on *A. trifoliata*; Shen et al. (2007) established and optimized a method for callus cultures in *A. trifoliata*, the results of which showed that the leaves comprise the best explants, and low pH and NAA 1.0 mg/L+2,4-D 4.0 mg/L+KT 1.0 mg/L can improve the induction efficiency of callus. In addition, a rapid method of micropropagation based on stems with leaf buds has been established in *A. trifoliata*; both induction and rooting rates were > 80% in the optimized medium. This method can shorten the nursery cycle of the seedlings (Wu et al., 2015). In addition to organogenesis, somatic embryogenesis is an important method of rapid propagation. Zou et al. (2019a) developed a simple and effective protocol for recurrent somatic embryogenesis in *A. trifoliata*, based on immature zygotic embryos from the roots. The highly efficient recurrent somatic embryogenesis implies a potential approach to large-scale rapid and mass propagation and promotes the genetic improvement and breeding process of *A. trifoliata*.

**Table 4:** Rapid micropropagation in *Akebia* genus.

**Species**	**Explant**	**Medium and PGRs**	**Condition**	**Target**	**Efficiency**	**References**
*quinata*	Stem segments	Solid MS medium containing sucrose (3% w/v) and agar (0.9% w/v)	In the dark at 26 °C for 4–5 week	Callus cultures	-	Ikuta (1991)
*trifoliata*	Leave	MS medium with 4 mg/L 2,4-D, 1 mg/L NAA, and 1 mg/L Kin	pH was 5.8, the temperature was 25 °C, and the cultures were kept in darkness	Callus cultures	87.5%	Shen et al. (2007)
	Stem with leaf buds	Inducing medium: WPM+1.0 mg·L-1 6-BA+0.5 mg·L-1 IAA+2.0 mg·L-1 GA3; multiplying medium: WPM+3.0 mg·L-1 6-BA+0.1 mg·L-1 IBA; rooting medium was 1/2MS+1.0 mg·L-1 IBA+0.5 mg·L-1 NAA+1.0 mg·L-1 GA3,	Humid air at 26 °C. The lighting intensity 50–60 μmol·m-2·s-1, photoperiod (14 h)	Micropropagation	Inducing rate 81.27%; rooting rate: 82.18%	Wu et al. (2015)
	Immature zygotic embryo from root	Inducing medium: MS without PGRs, pH 5.8; Regeneration medium: MS with 0.5 mg/L (BA);	25 °C, 16 h photoperiod and a photon fux density of 45 μmol m−2 s−1.	Recurrent somatic embryogenesis	95.8%	Zou et al. (2019a)

*MS, Murashige-Skoog medium; WPM, McCown's Woody Plant Basic Medium; 2,4-D, 2,4-dichlorophenoxyacetic acid; NAA, 1-naphthylacetic acid; Kin, kinetin; 6-BA, 6-benzylaminopurine; IAA, 3-Indoleacetic acid; IBA, 3-Indolebutyric acid; A: GA3, gibberellin A3; PGRs, plant growth regulators*.

#### Biochemical and Molecular Processes During Fruit Ripening

The fruit is a part of the *Akebia* genus of plants, especially *A. trifoliata* and *A. quinata*, which is not only used in traditional Chinese medicine but also as a food product. The perishable nature of *Akebia* fruit limits its shelf life post-harvest. The storage time and quality may depend on harvest time, transportation, and storage conditions. A previous report found that *Akebia* fruits are climacteric, whereby the respiration intensity reached its peak approximately 3–5 days post-harvest. The same authors found that implementing a controlled temperature (<7°C), using a chemical preservative (NaSO_3_ 2%), and reducing the oxygen concentration (<3%) could effectively prolong the fruit preservation time (Cao et al., 2003). Fruit cracking and softening also affect fruit quality during ripening and storage (Singh et al., 2020). Recent studies have focused on fruit cracking and flesh softening, which are consequential of multiple cellular processes. Microscopic results suggest that the structure and texture of the cell wall changed significantly during the initial cracking stage, such as thinner and looser cell walls, fewer cell layers, and increased space between cells in the pericarp (Niu et al., 2020; Jiang et al., 2022). Although significant changes in physiology and biochemistry have been observed during fruit cracking in *A. trifoliata*, such as the depolymerization of covalently bound pectin and cellulose, disordered ROS (reactive oxygen species) homeostasis, decrease in minerals (K and Ca), degradation of starch, and water movement, these changes may be regulated by phytohormones through increased indole-3-acetic acid (IAA), salicylic acid, and jasmonic acid levels, as well as decreased cytokinin content (Jiang et al., 2022). Numerous mRNAs and proteins involved in cell wall metabolism, plant hormone regulation, ROS homeostasis, and stress response have been identified and may be associated with fruit cracking and flesh softening (Table 5). Additionally, extensive remodeling of the cell wall structure occurs during fruit ripening and the depolymerization of multiple polysaccharide networks through different cell wall-modified proteins (Nishiyama et al., 2007). Changes in PEs, PL, PG, β-GAL, and PAE at the transcriptional and translational levels during fruit cracking and flesh softening in *A. trifoliata* could promote cell wall polysaccharide modification and subsequent degradation of pectin polysaccharides, and XTHs, CEL, BXL, and ASD may participate in cellulose disassembly matrix and hemicellulose degradation in the cell wall. Expansin was first identified as a cell wall-loosening protein involved in regulating a variety of plant processes, such as the softening of fruit (Jin et al., 2006). These processes of cell wall metabolism have also been observed in the fruits of other plants (Brummell and Harpster, 2001; Vicente et al., 2007; Wang et al., 2018). PRXs are also important oxidoreductase enzymes and are tightly associated with cell wall rearrangement because ROS can initiate the disassembly of cell wall polysaccharides and accelerate fruit ripening (Berni et al., 2019). In addition to PRXs, the high expression of chitinase, endochitinase, cysteine protease, and thaumatin-like proteins may also be involved in ROS accumulation during fruit ripening in *Akebia* spp. Moreover, certain proteins, such as PL, PE, β-GAL, and PRX, are regarded as hub nodes in the PPI network and might interact with other target proteins involved in fruit ripening and softening (Niu et al., 2021).

**Table 5:** Different expressional gene and different abundant protein during fruit cracking and soften in *A*. trifoliata^*^ .

**Protein**	**Gene**	**Tissue**	**Biological process**	**Function**	**Fruit cracking**	**After cracking**
Polygalacturonase	*PG*	Pericarp	Cell wall metabolism	Hydrolytic cleavage unesterified pectin	Up-regulation	Up-regulation
		Flesh			Down-regulation	Up-regulation
	*PG3*	Pericarp			Down-regulation	Up-regulation
Pectinesterase	*PE*	Pericarp		Removal of methyl groups from esterified pectin	Up-regulation	Up-regulation
		Flesh			Down-regulation	Up-regulation
	*PE2*	Flesh			Down-regulation	Up-regulation
	*PE3*	Flesh			Up-regulation	Up-regulation
Pectate lyase	*PL*	Pericarp		Eliminative cleavage of pectate,	Up-regulation	Up-regulation
		Flesh			Down-regulation	Up-regulation
	*PL2*	Flesh			Up-regulation	Up-regulation
Pectin acetylesterase	*PAE*	Flesh		Hydrolysis of acetyl esters of pectin	Down-regulation	Up-regulation
β-galactosidase	*β-GAL1*	Pericarp		Removal of galactosyl residues increased from pectin	Down-regulation	Down-regulation
		Flesh			Down-regulation	Up-regulation
	*β-GAL2*	Pericarp			Down-regulation	Up-regulation
		Flesh			Down-regulation	Up-regulation
Expansin	*EXP*	Pericarp		Wall stress relaxation and irreversible wall extension	Down-regulation	Down-regulation
	*EXP1*	Flesh		Cell wall loosen	Up-regulation	Up-regulation
	*EXP2*	Flesh			Down-regulation	Up-regulation
endoglucanase	*CEL*	Pericarp		Cellulose matrix disassembly	Down-regulation	Up-regulation
	*CEL1*	Flesh			Down-regulation	Up-regulation
	*CEL2*	Flesh			Down-regulation	Up-regulation
Furostanol glycoside 26-O-betaglucosidase	*F26G*	Pericarp		Starch and sucrose metabolism	Up-regulation	Up-regulation
Alpha/beta hydrolase	*α-HY*	Pericarp			Up-regulation	Down-regulation
Glucan endo-1,3-betad-glucosidase	*ENDOB*	Pericarp			Down-regulation	Up-regulation
Beta-D-xylosidase	*XYL*	Pericarp			Up-regulation	Down-regulation
Cellulose synthase-like protein	*CSLG*	Pericarp		Cellulose synthesis	Up-regulation	Up-regulation
β-xylosidase	*BXL*	Flesh		Hemicellulose degradation	Down-regulation	Up-regulation
α-arabinofuranosidase	*ASD*	Flesh			Down-regulation	Up-regulation
Inactive beta-amylase	*BAM*	Flesh		Starch degradation	Up-regulation	Up-regulation
Beta-fructofuranosidase	*β-FRU*	Flesh		Sugar accumulation	Up-regulation	Up-regulation
Peroxidase	*PRX*	Pericarp	ROS homeostasis	Removal of hydrogen peroxide	Down-regulation	Up-regulation
		Flesh		ROS-scavenging capacity	Up-regulation	Up-regulation
	*PRX2*	Pericarp			Down-regulation	Up-regulation
	*PRX3*	Pericarp			Down-regulation	Up-regulation
	*PXR5*	Pericarp			Down-regulation	Up-regulation
Cysteine protease	*RD21*	Flesh		Response to the accumulation of ROS	Down-regulation	Up-regulation
Thaumatin-like proteins	*TLP*	Flesh		Response to the accumulation of ROS	Up-regulation	Up-regulation
Heat shock proteins	*HSP20*	Flesh	Biotic and abiotic stresses	Stress response	Up-regulation	Down-regulation
	*HSP20–2*	Flesh			Up-regulation	Down-regulation
	*HSP20–3*	Flesh			Up-regulation	No change
Cinnamyl-alcohol dehydrogenase	*CAD*	Pericarp	Phenylpropanoid biosynthesis	Controls mechanical strength	Up-regulation	Down-regulation
Cinnamoyl-CoA reductase	*CCR*	Flesh			Down-regulation	Up-regulation
4-coumarate-COA-ligase	*4CL*	Pericarp			Down-regulation	Down-regulation
Shikimate O-Hydroxycinnamoyltransferase	*HCT*	Pericarp			Up-regulation	Up-regulation
Auxin efux carrier	*AEC*	Pericarp	Phytohormone		Up-regulation	Down-regulation
Gibberellin-regulated protein	*GRP*	Flesh			Down-regulation	Up-regulation
NAC domain-containing protein	*NAC/NAC-like*	Pericarp	Transcriptional control		Down-regulation	Up-regulation
Transcription factor bHLH66	*bHLH*	Pericarp			Up-regulation	Up-regulation
Dirigent protein	*DIR2*	Pericarp	Others		Up-regulation	Up-regulation
Ripening-related protein	*grip22*	Flesh			Up-regulation	Up-regulation

* *Data sourced from Niu et al. (2020, 2021)*.

#### Characterization of Genome and Fatty Acid Synthesis

Recently, significant progress was made when the whole genome of *A. trifoliata* subsp. australis was decoded by a Chinese research team (Huang H. et al., 2021). A high-quality chromosome-level genome sequence of *A. trifoliata* subsp. australis has been reported, and a *de novo* genome assembly of 682.14 Mb was generated with a scaffold N50 of 43.11 Mb. A KEGG enrichment analysis showed that the sesquiterpenoid, triterpenoid, and monoterpenoid biosynthesis pathways were significantly enriched in the expanded genes. Twenty-four β-amyrin synthase-like (*Atr*β*-AS*) genes that can convert oxidosqualene to β-amyrin in plants were found in the *Akebia* genome. These expanded genes may account for the content and diversity of oleanane-type saponins in *A. trifoliata* (Zhao et al., 2015). In addition, UDP-glucuronosyl, UDP-glucosyltransferase, and chrome P450 gene families have also been expanded and contracted, and some of these genes are believed to be involved in downstream reactions in the biosynthesis of saponins, such as cytochrome P450-dependent hydroxylation/oxidations and several glycosyl transfer reactions (Achnine et al., 2005). The molecular foundation for unsaturated fatty acid biosynthesis was also revealed by the transcriptome and metabolic profiles. The FA and TAG biosynthetic pathways were reconstructed, and the expression pattern of key genes during the development of seeds was uncovered, such as acetyl-CoA carboxylase (ACCase), fatty-acid synthase (FAS), three acyl-ACP thioesterases (FATA/B), and three long-chain acyl-CoA synthetases (LACS), which play an essential role in fatty-acid biosynthesis, chain termination, FA transport, and conversion. Comparative genomics analyses may also suggest a molecular basis of specific FA profiles in *A. trifoliata* because of the expanded gene family on linoleic acid and α-linolenic acid metabolism, UFA, and FA biosynthesis. Decoding the genome of *A. trifoliate* provides crucial insight into the formation of economic traits and could provide fundamental information for the genetic improvement of *Akebia* plants.

#### Breeding and Potential Directions

The utilization of *Akebia* plants has a long history, such as in herbal medicine and wild fruit. The breeding of *Akebia* spp. is still in its infancy based on discovery and selection from wild resources. However, some gardens have been developed by amateur horticulturists, and the domestication and breeding of *Akebia* have also taken place in Japan, such as developing clones with high tolerance to powdery mildew (Nobuko et al., 2000). In China, a few previous studies have established the systematic breeding of *Akebia* spp. The development of *Akebia* plants from seed to fruit takes about 3 years, and a cycle of selection may take 5 years for an excellent individual. Compared with established crops, this process remains time-consuming, but it is still better than that of most woody plants. A three-generation recurrent selection population was built based on fruit weight and pulp (Zou et al., 2018). The selection of economic traits and genetic analysis of *A. trifoliata* has been reported recently Zou et al. (2019b). The latter studies indicate that recurrent selection is an effective breeding method, and the selection of multiple targeted traits is also feasible. In addition, certain superior lineages could be developed with high single fruit weight and edible rate in *A. trifoliata*. *Akebia* species have many excellent economic traits and properties, which indicate that they could be developed as a potential new oil crop and fruit, in addition to providing traditional herbal medicines and gardening plants. Certain studies have also found that extraction from the waste of *Akbeia* plants, such as the pericarp, can be used to develop additional beverages and new medicinal materials (Yu et al., 2019; Cai et al., 2020).

**Figure 3:**
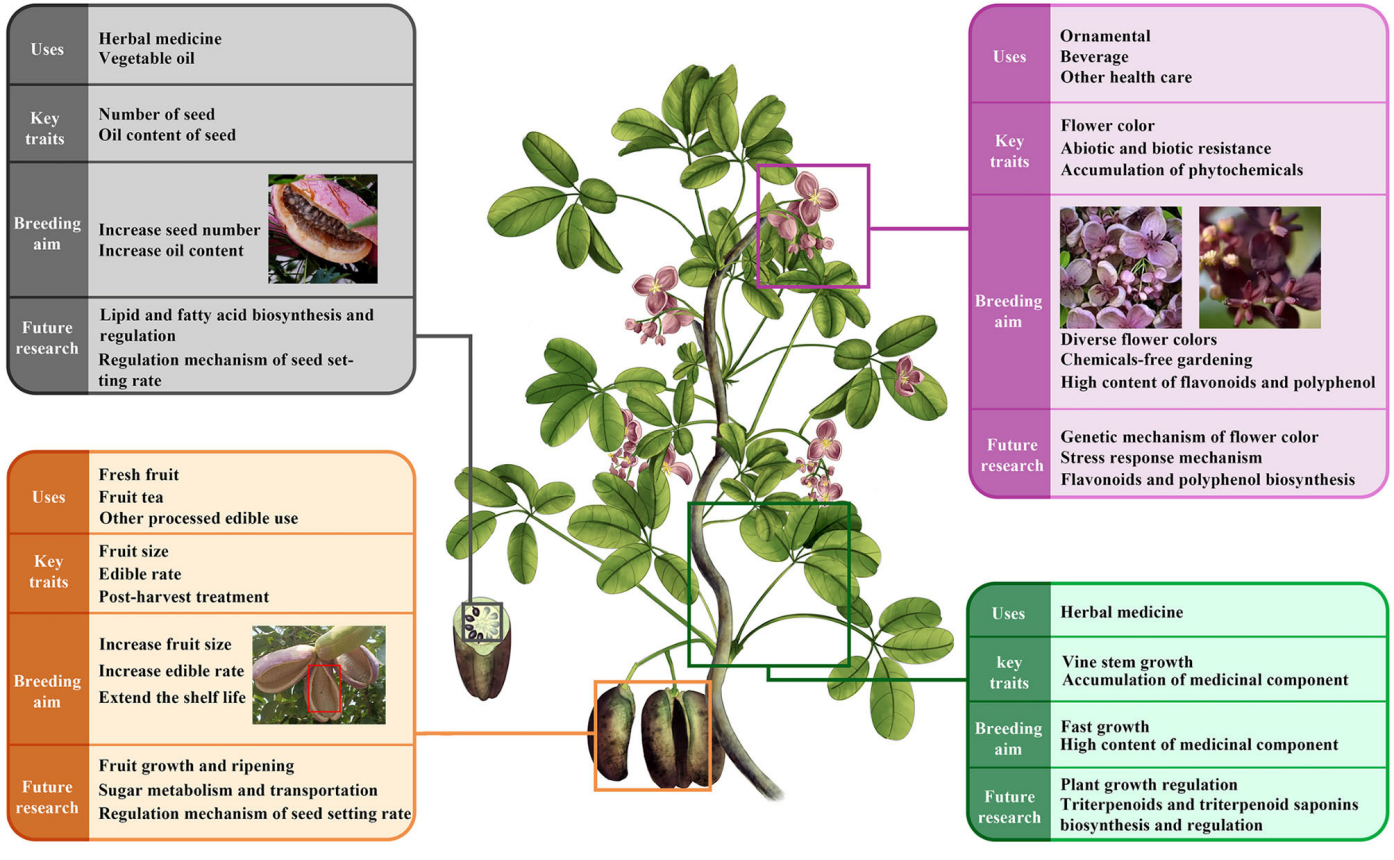
Potential uses, key traits, and breeding aim of *Akebia* species.

However, the domestication and breeding of *Akebia* spp. still requires much development. It is possible to accelerate this process with the help of modern biological techniques based on new multidisciplinary knowledge. Phytochemicals are important sources of medicine and nutrition for humans and are of great significance to human survival and health (Bjorkman et al., 2011; Barbieri et al., 2017). Most of *Akebia's* herbal medicines come from wild sources. This may be unsustainable given the increasing need for human society. Therefore, systematic domestication and cultivation provide a solution to balance the supply and demand of *Akebia* products. Faster growth and accumulation of medicinal components in vine stems could be one of the directions for producing genuine herbal medicines in *Akebia* plants. Thus, we may need to pay more attention to mechanisms regarding growth regulation and triterpenoid synthesis. *Akebia* fruit is edible and has a preferential taste, however, the ER is low (approximately 30%) because of the many seeds present and thick peels. Improving SFW and ER is needed, and future studies may need to focus on the development and maturity of the fruit, post-harvest physiology, regulation technology, and control of setting percentages. In contrast, the production of vegetable oil requires more seeds and higher oil content. Anthocyanins are abundant in the flowers of *Akebia* plants, such as cyanidin and delphinidin, which provide the material basis and possibility for breeding garden varieties of different colors and for understanding the genetic basis of color variation facilities for parental selection during *Akebia* plant breeding (Figure 3).

## Conclusions

*Akebia* species comprise subtropical vines with great economic potential for food, nutrition, and health. The stems and seeds of *Akebia* are sourced as traditional herbal medicines; the fruit is edible and has a preferential taste, and its extract can be used to produce health care products and new medical materials. In this review, we introduced its botanical and ecological characteristics and the status of its genetic resources. The assessment of its genetic resources suggests that the wild population maintains a relatively high level of genetic diversity and low genetic differentiation, and abundant phenotypic variation, such as among fruit, and provides fundamental material for future breeding. Nevertheless, it is necessary to strengthen resource conservation and the sustainable use of *Akebia*, facing land-use and climate changes. We also summarized the progress of phytochemical studies and biotechnology in *Akebia*. A rich variety of phytochemicals in *Akebia* forms the basis of development utilization; numerous triterpenoids and triterpenoid saponins have been isolated from the stems, fruit, and seeds of *Akebia spp*., which are beneficial for the promotion of human health. In addition, the fruits and seeds contain redundant nutrient substances and microelements, such as sugars, proteins, amino acids, and fatty acids. The ratios of saturated, monounsaturated, and polyunsaturated fatty acids meet the WHO recommendation standards for edible oil. In addition, *Akebia* spp. are not model plants, but some progress has been made in biotechnology research. A series of micropropagation techniques have been established based on organogenesis and embryogenesis in *Akebia*, which could provide new knowledge and methods for the rapid propagation and construction of genetic transformation systems. Since a high-quality genome of *A. trifoliata* has been assembled and annotated, the genetic basis of secondary metabolites and fatty acid synthesis has also been preliminarily understood at the transcriptional and metabolic levels. In addition, several related genes and TFs involved in fruit cracking and pulp softening have been explored by comparative transcriptome analysis. These genes and TFs are involved in cell wall metabolism, ROS homeostasis, stress responses, and phytohormone regulation. Finally, in this review, we introduced the status of *Akebia* breeding and propose potential directions for future research.

## Author Contributions

PH contributed to analyzing literature and writing the draft manuscripts. FZ and CL contributed to data collection and drawing the figures. FL and DZ contributed to revising the manuscript. BL and YZ conceived the study and revised the manuscripts. All authors contributed to the article and approved the submitted version.

## Funding

This work was funded by National Center for Forestry and Grassland Genetic Resources, China (NCFGGR2021); Development Center for Science and Technology, National Forestry and Grassland Administration, China (KJZXXP202217); Establishment and Managemnet Program of National Forest Germplasm Resources Bank for Gardens Tree Specie (2022–2023).

## Conflict of Interest

The authors declare that the research was conducted in the absence of any commercial or financial relationships that could be construed as a potential conflict of interest.

## Publisher's Note

All claims expressed in this article are solely those of the authors and do not necessarily represent those of their affiliated organizations, or those of the publisher, the editors and the reviewers. Any product that may be evaluated in this article, or claim that may be made by its manufacturer, is not guaranteed or endorsed by the publisher.
